# Exercise Physiology Impairments of Patients With Amyotrophic Lateral Sclerosis: Cardiopulmonary Exercise Testing Findings

**DOI:** 10.3389/fphys.2022.792660

**Published:** 2022-03-14

**Authors:** Ji He, Jiayu Fu, Wei Zhao, Chuan Ren, Ping Liu, Lu Chen, Dan Li, Lequn Zhou, Lu Tang, Xiangyi Liu, Shan Ye, Xiaolu Liu, Yan Ma, Yixuan Zhang, Xinran Ma, Linjing Zhang, Gaoqi Zhang, Nan Li, Dongsheng Fan

**Affiliations:** ^1^Department of Neurology, Peking University Third Hospital, Beijing, China; ^2^Beijing Municipal Key Laboratory of Biomarker and Translational Research in Neurodegenerative Diseases, Beijing, China; ^3^Department of Cardiology, Peking University Third Hospital, Beijing, China; ^4^Physical Examination Center, Peking University Third Hospital, Beijing, China; ^5^Clinical Epidemiology Research Center, Peking University Third Hospital, Beijing, China

**Keywords:** exercise physiology evaluation, cardiopulmonary exercise testing, amyotrophic lateral sclerosis, prognosis, exercise capacity

## Abstract

**Background and Objective:**

In amyotrophic lateral sclerosis (ALS), progressive weakness significantly limits the ability to exercise. However, measurements of the impaired exercise function and their practical value to assess disease progression in ALS are scarce. Cardiopulmonary exercise testing (CPET) is a non-invasive accurate method used to comprehensively quantify exercise physiology in a variety of diseases. This study aimed to evaluate the clinical value of CPET and to explore its association with disease severity and prognosis prediction in ALS.

**Methods:**

A total of 319 participants were enrolled in this 3-year prospective study. After strict quality control, 109 patients with ALS and 150 age- and sex-matched healthy controls were included with comprehensive clinical assessment and follow-ups. The incremental ramp protocol for symptom-limited CPET was applied in both groups. The exercise physiology during peak effort exercise was systematically measured, including the overall aerobic capacity of exercise (VO_2_ peak) and the respective capacity of the exercise-involved organs [cardiac response (heart rate peak—HR peak), ventilatory efficiency (VE/VCO_2_ slope), breathing economy (VE/VO_2_ peak), and other relevant parameters]. Disease severity and progression were evaluated using recognized scales. Survival was monitored with regular follow-ups every 6 months.

**Results:**

Decreased exercise capacity (VO_2_ peak < 16 ml/kg/min) occurred more frequently in patients with ALS than in controls (44.95% vs. 9.33%, *p* < 0.01). In patients with ALS, the average VO_2_ peak (16.16 ± 5.43 ml/kg/min) and HR peak [135 (112–153) bpm] were significantly lower (*p* < 0.01) than in controls [22.26 ± 7.09 ml/kg/min; 148 (135–164) bpm], but the VE/VCO_2_ slope was significantly higher [28.05 (25.03–32.16) vs. 26.72 (24.37–29.58); *p* = 0.03]. In patients with ALS, the VO_2_ peak and HR peak were significantly correlated with disease severity and progression scores (*p* < 0.05). Survival analyses revealed the VO_2_ peak and HR peak as protective indicators while the VE/VO_2_ peak as a detrimental indicator for the prognostic prediction in ALS (HR = 0.839, *p* = 0.001; HR = 0.967, *p* < 0.001; HR = 1.137, *p* = 0.028, respectively).

**Conclusion:**

Our prospective study quantified the significantly decreased exercise capacity in ALS through non-invasive CPET. The impaired VO_2_ peak and HR peak closely correlated with disease severity and independently predicted a worse prognosis. Our findings identified the clinical value of CPET as an objective indicator of disease progression in ALS.

## Introduction

Amyotrophic lateral sclerosis (ALS) is a fatal neurodegenerative disorder characterized by progressive dysfunction in the motor system (van Es et al., 2017). The impairments are irreversible, which start from the weakness of the first affected site to paralysis, dysphagia, and eventually respiratory failure. Thus, patients are significantly limited in their ability to exercise (Chen et al., 2015). Recent identifications of pathophysiological mechanisms of ALS potentially provide new insights, including autonomic dysregulation for heart rate (HR) variability and hypermetabolism for respiratory decompensation (Hardiman et al., 2017; Ahmed et al., 2018). Moreover, a growing body of evidence has identified consistent mitochondrial abnormalities in neuromuscular tissues of patients with ALS and mouse models (Dupuis et al., 2004; Al-Sarraj et al., 2014; Jankovic et al., 2021). Though the pathogenesis of ALS is different from the metabolic myopathy, such as mitochondrial disease, dysfunction of mitochondria has been linked to muscle weakness in ALS: (1) the deposition of pathological protein of ALS such as TAR DNA-binding protein 43 (TDP-43) impairs the mitochondrial structure, which further induces death of muscular cells through devastating mitophagy, excitotoxicity, and oxidative stress (Kodavati et al., 2020). The consequent loss of actin and myosin filaments limits effective contractions of single-muscle fibers as the sliding filament theory, functionally manifesting as muscle weakness in patients (Miller et al., 2014). (2) Adenosine triphosphate (ATP) is the source of energy used to power muscle contraction. The intracellular-stored ATP is not enough for continuous muscular activities, hence the replenishment of ATP through mitochondrial respiration is substantial to high-intensity muscle movements (Baker et al., 2010). Overexpressing mutant TDP-43 of the ALS model decreases mitochondrial complex I activity, which further decreases oxygen utilization and ATP production *via* defective oxidative phosphorylation (Huang et al., 2020). The oxidative stress and inflammation in ALS aggravate the decrease in ATP regeneration and cause depletion of energy (Obrador et al., 2021). Thus, the insufficient energy supply induced by dysregulated mitochondria is linked to muscle fatigue and weakness in ALS. (3) In TDP-43 mouse models, distinct changes in mitochondrial dynamic and aggregation are reported (Kodavati et al., 2020). Mutated TDP-43 affects mitofusin 1 and 2 levels, causing fragmented mitochondrial morphology and muscle atrophy that further decreases muscular strength (Wang et al., 2013). (4) Dysregulated mitochondria are associated with dysfunction of ion channels (including Na^+^, K^+^, and Ca^2+^) in ALS (LoRusso et al., 2019). The abnormalities disturb the excitation-contraction coupling and decrease the excitability of muscle fibers that further induce muscle fatigue in patients (Allen et al., 2008). Thus, mitochondrial dysfunction has emerged as one of the significant reasons for the exercise limitation of ALS (Angelini and Siciliano, 2021). Cross-sectional studies and pilot trials of small size have revealed significant exercise intolerance in ALS (Sanjak et al., 1987; Siciliano et al., 2002; Mezzani et al., 2012; Braga et al., 2018). Further studies with more patients to explore its clinical value for the assessment of disease progression are required. The current methods for disease evaluation are mainly based on the disability degree, represented by the revised ALS Functional Rating Scale (ALSFRS-R) and the King’s College Staging System (KCSS) (Cedarbaum et al., 1999; Roche et al., 2012). Thus, the exploration of direct indicators for exercise capability associated with disease severity and prognosis could provide a novel prospective for disease evaluation (Sun et al., 2020). Detailed assessments of the systemic physiological responses to exercise could also give insights into potential aerobic rehabilitation in ALS.

Cardiopulmonary exercise testing (CPET) is recommended as the gold standard for objectively evaluating exercise physiology by the European Association for Cardiovascular Prevention and Rehabilitation and the American Heart Association (EACPR/AHA) (Guazzi et al., 2012, 2016). It can provide a comprehensive assessment in multiple relevant physiological systems, including the cardiovascular, pulmonary, and musculoskeletal systems (Fan and Jia, 2020). To quantitatively analyze exercise physiology, CPET non-invasively measures extensive variables reflecting different physiological responses under peak exercise stress (Arena and Sietsema, 2011). Oxygen uptake at peak exercise (VO_2_ peak) is well recognized to represent the aerobic exercise capacity for the integrated function of the whole-body exercise capability. Other parameters separately represent the singular function of the system-targeted exercise physiology (i.e., HR recovery for the cardiovascular system, VE/VCO_2_ slope for the pulmonary system) (Taivassalo et al., 2003; Crisafulli et al., 2018; Opina et al., 2019). The clinical value of CPET in cardiovascular and pulmonary diseases has been firmly established for preoperative evaluation and prognosis assessment (Grant et al., 2015; Guazzi et al., 2017). Recent studies have identified the reliable use of CPET in neurological diseases, including metabolic myopathies, stroke, multiple sclerosis, and Huntington’s disease; however, there is a lack of application of CPET for ALS so far (Jeppesen et al., 2003; Taivassalo et al., 2003; Ciammola et al., 2011; Heine et al., 2014, 2016; Fan and Jia, 2020). As a non-invasive tool that allows for quantitative assessment, CPET could supplement previous evaluations of functional disability and further provide a comprehensive profile of exercise physiology impairments for ALS.

We aimed to evaluate the exercise physiology impairments using CPET in patients with ALS compared with age- and sex-matched healthy controls. In addition, we explored their clinical values to assess disease severity and prognosis of ALS from a clinical physiology perspective in our prospective cohort.

## Materials and Methods

### Study Design and Participants

**FIGURE 1:**
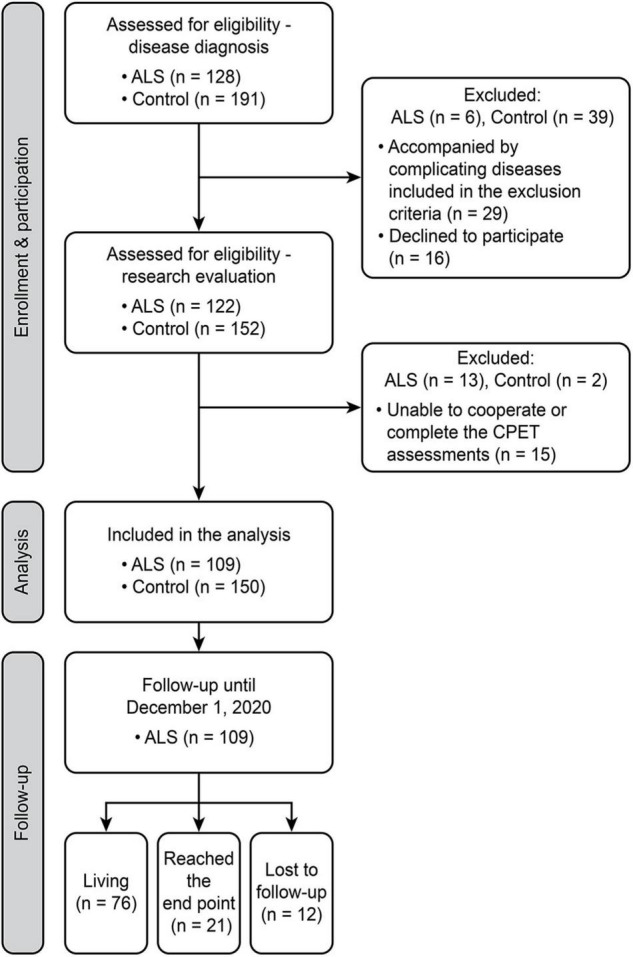
Flowchart of the study design. A schematic summarizing the number of individuals during enrollment, participation, inclusion, and follow-up. ALS, amyotrophic lateral sclerosis; CPET, cardiopulmonary exercise testing.

This prospective study was conducted between September 1, 2017, and December 1, 2020, at the Peking University Third Hospital (PUTH), Beijing, China. A total of 128 consecutive patients with ALS who fulfilled the revised El Escorial criteria for probable or definite ALS were invited to participate in the study and were enrolled at the time of diagnosis. The exclusion criteria for patients included (1) concomitant presence of cardiopulmonary, endocrine, or neurological disorders other than ALS; (2) cardiovascular impairments noted on structural ultrasonic cardiography and electrocardiography; (3) pulmonary impairments noted on structural X-ray; (4) cognitive impairment or psychiatric disorders; (5) family history of ALS; and (6) inability to complete the cycling test of CPET. Moreover, 191 age- and sex-matched healthy controls were consecutively enrolled from volunteers in the Physical Examination Center, PUTH. These healthy controls had no cardiopulmonary, endocrine, or neurological disorders, no family history of any known inherited disease, no cognitive or psychiatric disorders, and no abnormalities in routine physical examination, and they were able to complete CPET. After a strict screening of the aforementioned comorbidities and agreeing to cooperate with the study, 109 patients with ALS and 150 controls were finally included. A total of 19 patients were excluded because of chronic obstructive pulmonary disease (*n* = 1), diabetes (*n* = 1), coronary heart disease (*n* = 2), hypertension (*n* = 1), refusal to the test (*n* = 1), and inability to complete CPET assessment with the protocol (*n* = 13). In total, 41 controls were excluded due to diabetes (*n* = 8), chronic obstructive pulmonary disease (*n* = 3), coronary heart disease (*n* = 7), hypertension (*n* = 6), refusal to the test (*n* = 15), and inability to complete CPET assessment with the protocol (*n* = 2). The institutional Ethics Committee of PUTH approved this study. Written informed consent was obtained from each participant. Study enrollment, participation, analysis, and follow-up are illustrated in Figure 1.

### Clinical Assessment

At enrollment, detailed demographic and clinical information was recorded, and regular laboratory tests were conducted as a standardized routine for patients with ALS at PUTH (Supplementary Table 1). The collected data included age, sex, medical history, site of onset, age of onset, disease duration, genetic variants, cognitive assessments, blood glucose, blood lipids, and other relevant data as reported previously (Chen et al., 2015; He et al., 2020). We recorded the results of ALSFRS-R and KCSS, as well as forced vital capacity (FVC), to functionally evaluate disease severity (Ferraro et al., 2016). FVC was measured in the sitting position using computer-based spirometry (Chest, HI-101, Japan). The test was performed with the same device and by the same technician with the standard protocol. The best of three satisfactory and consistent expiratory maneuvers (each obtained after a maximal inspiratory effort) and the predicted values (%) were used for analyses. The impairment of upper motor neuron (UMN) and lower motor neuron (LMN) signs was graded using a modified Ravits Scale (Ravits et al., 2007). The rate of disease progression was recorded as ΔFS = (48-[ALSFRS-R])/disease duration from symptom onset to the CPET assessment. Serum neurofilament light chain (sNfL) concentrations were tested using Simoa NfL assays by Quanterix (Lexington, MA, United States) (Benatar et al., 2020). Death and tracheotomy were defined as the endpoint events for survival. Follow-up evaluations were conducted by telephone or clinical visits every 6 months to assess survival and record parameters for disease progression of ALS, including ALSFRS-R, body mass index (BMI), affected sites, and other details (Supplementary Table 1).

### Cardiopulmonary Exercise Testing

Symptom-limited CPET was performed according to published guidelines using the standardized Ramp protocol for the cycling test (Balady et al., 2010). Ventilatory expired gas analysis was performed using the ULTIMACardio2 gas exchange analysis system (Medgraphics Corp, United States) with the detailed procedures reported previously (Liu et al., 2020). Minute ventilatory data were obtained at rest and during the incremental workload exercise test based on real-time gas exchange measurements using the open-circuit spirometry testing system [including oxygen consumption (VO_2_), carbon dioxide production (VCO_2_), and minute ventilation (VE)]. A standard 12-lead electrocardiogram was obtained, and blood pressure was measured at rest, each minute during exercise, and for at least 5 min during the recovery phase. Standardized procedures were conducted for equipment calibration, and all tests were carried out by trained clinical exercise physiologists. The detailed protocol of the CPET assessment was as follows: the test started with 3 min of rest, followed by a 3-min warm-up at 0 W, and continued to cycle with an increased workload of 10 W every minute until volitional fatigue. A uniform speed of 60–70 r/min was encouraged to maintain when cycling. The total duration of the cycle test typically was 10–15 min. A respiratory exchange ratio (RER) of ≥ 1.0 was used as an objective indicator of peak effort for exercise (Guazzi et al., 2012, 2016).

The major parameter in this study was the VO_2_ peak achieved at peak exercise, which reflects the overall level of exercise capacity affected by the cardiovascular, pulmonary, and muscular function. Decreased exercise capacity was defined by a VO_2_ peak < 16 ml/kg/min, as reported previously (Arena and Sietsema, 2011; Guazzi et al., 2017). Other parameters were divided according to the individually involved system as reported by previous studies. For cardiovascular function, HR, systolic blood pressure (SBP), and diastolic blood pressure (DBP) at rest and peak exercise, as well as HT recovery (peak HR- 1 min after exercise HR), were recorded (Crisafulli et al., 2018). For pulmonary function, ventilation per carbon dioxide output slope (VE/VCO_2_ slope) and breathing reserve (BR) were calculated (Finocchiaro et al., 2015; Opina et al., 2019). For muscular function, the oxygen cost (ΔVO_2_/ΔWork-Rate slope) was reported to represent the ability of muscles to extract and use oxygen (Taivassalo et al., 2003; Noury et al., 2020). The peak ventilatory equivalent for oxygen (VE/VO_2_ peak) represented a comprehensive indicator of breathing economy.

### Statistical Analysis

Qualitative data are reported as numbers and percentages (%) of cases, and the chi-square test was used for comparisons between groups. Quantitative data were first tested to determine the normality of the distribution. Normally distributed data are reported as means (standard deviations), variables between groups were compared using Student’s *t*-test or one-way ANOVA, and correlations were assessed using Pearson’s analysis. Non-normally distributed variables are shown as medians (first quartile, third quartile), variables between groups were compared using the Mann-Whitney *U*-test or Kruskal-Wallis test, and correlations were assessed using Spearman’s analysis. Subsequently, a multiple linear regression analysis was performed to assess the adjusted association of disease-related variables with the VO_2_ peak. Survival curves were estimated using Kaplan-Meier analysis with the log-rank test. Cox regression model adjusted for covariates, including the age of onset, sex, BMI, and site of onset, was performed. The censoring date for survival data was December 1, 2020. Statistical analysis was performed with SPSS 20.0 software (SPSS, Chicago, United States). The results were considered statistically significant at *p* < 0.05.

## Results

### Demographic and Clinical Features of Amyotrophic Lateral Sclerosis and Controls

Our study initially enrolled 319 participants, and 60 were excluded after a strict screening of medical history, assessment of cardiopulmonary function, and cooperation with the CPET measurements (Figure 1). A total of 259 participants were eventually included. Table 1 shows the baseline characteristics of the 109 patients with ALS and the 150 matched controls. The ALS and control groups did not differ significantly in age, sex, or BMI. Regarding the clinical features of ALS, the median age of onset (interquartile range, IQR) was 53 (42–61) years, with median disease duration (IQR) of 12 (8–17) months. Of the 109 patients with ALS, 19 had a bulbar onset (17.43%) and 90 had a spinal onset (82.57%). Most patients were in the early phase of the disease, consisting of 53 in KCSS 1 (48.62%) and 37 in KCSS2 (33.94%). The median ALSFRS-R (IQR) score was 43 (39–45).

**TABLE 1:** Baseline characteristics of ALS and controls.

	ALS, *n* = 109	Control, *n* = 150	*p*-value
**Demographics**			
Age, years	52.74 (11.62)	51.39 (11.47)	0.35
**Sex, no. (%)**			0.55
Male	71 (65.14%)	103 (68.67%)	
Female	38 (34.86%)	47 (31.33%)	
BMI, kg/m^2^	23.83 (3.35)	24.36 (3.06)	0.19
**Disease characteristics**			
Age of onset, years	53 (42–61)		
**Site of onset, no. (%)**			
Bulbar	19 (17.43%)		
Spinal	90 (82.57%)		
Disease duration, months	12 (8–17)		
**KCSS**			
1	53 (48.62%)		
2	37 (33.94%)		
3	12 (11.01%)		
4	7 (6.42%)		
ALSFRS-R	43 (39–45)		
FVC,% of predicted	81 (71–93)		
UMN	6 (3–8)		
LMN	3 (2–4)		
NfL, pg/ml	70.30 (33.00–101.00)		
ΔFS	0.43 (0.25–0.71)		
Use of riluzole, no. (%)	71 (65.14%)		

*Data are presented as the means (SD), medians (IQR), or n (%). ALS, amyotrophic lateral sclerosis; BMI, body mass index; KCSS, King’s College Staging System; ALSFRS-R, ALS Functional Rating Scale-Revised; FVC, forced vital capacity; UMN, upper motor neuron; LMN, lower motor neuron; NfL, neurofilament light chain; ΔFS, (48-[ALSFRS-R])/disease duration from symptom onset to the CPET assessment; IQR, interquartile range.*

### Comparison of the Cardiopulmonary Exercise Testing Assessment in Amyotrophic Lateral Sclerosis and Controls

**FIGURE 2:**
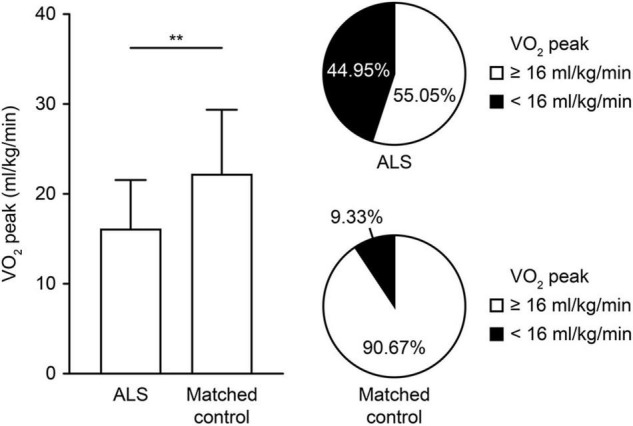
Comparison of exercise capacity measured by the CPET in controls and patients with ALS. The degree and proportion of impaired exercise capacity are depicted in controls and patients with ALS. The VO_2_ peak (ml/kg/min) represents the exercise capacity, and the impaired exercise capacity is defined by a VO_2_ peak < 16 ml/kg/min. **p < 0.01. ALS, amyotrophic lateral sclerosis; CPET, cardiopulmonary exercise testing; VO_2_ peak, oxygen uptake at peak exercise.

For the exercise capacity (VO_2_ peak) reflecting the whole-body system, patients with ALS showed a significant impairment both in prevalence and degree compared with the strictly matched controls (Figure 2). Impaired exercise capacity (VO_2_ peak < 16 ml/kg/min) was present in 49 (44.95%) patients with ALS and 14 (9.33%) controls, which showed significant differences in distribution (*p* < 0.01). Consistently, the VO_2_ peak was significantly lower in patients with ALS than in controls [Table 2; 16.16 (5.43) vs. 22.26 (7.09); *p* < 0.01]. Several parameters reflecting relative impairment regarding single systems also showed significant differences (Table 2). For the cardiovascular system, patients with ALS had a significantly increased HR at rest while a decreased HR at peak exercise (*p* = 0.01 and *p* < 0.01, respectively). However, no difference was shown in HR recovery between the two groups. For the pulmonary system, a significant increase was reported in VE/VCO_2_ slope in patients with ALS, implying limited ventilatory efficiency (*p* = 0.03). However, no significant differences in the parameters reflecting impairment in the muscular system were observed between the two groups.

**TABLE 2:** CPET assessments of patients with ALS and controls.

	ALS, *n* = 109	Control, *n* = 150	*p*-value
**Overall exercise capacity**			
VO_2_ peak, ml/kg/min	16.16 (5.43)	22.26 (7.09)	<0.01
VO_2_ peak ≥ 16 ml/kg/min, no. (%)	60 (55.05%)	136 (90.67%)	<0.01
VO_2_ peak < 16 ml/kg/min, no. (%)	49 (44.95%)	14 (9.33%)	
**Cardiovascular function**			
HR rest, beats/min	86 (78–97)	83 (73–90)	0.01
SBP rest, mm Hg	128 (118–138)	122 (113–138)	0.05
DBP rest, mm Hg	79 (74–86)	78 (70–86)	0.18
HR peak, beats/min	135 (112–153)	148 (135–164)	<0.01
SBP peak, mm Hg	165 (142–190)	166 (145–191)	0.79
DBP peak, mm Hg	86 (80–96)	83 (77–90)	0.05
HR recovery, beats/min	23 (17–31)	25 (19–31)	0.12
**Pulmonary function**			
VE/VCO_2_ slope	28.05 (25.03–32.16)	26.72 (24.37–29.58)	0.03
BR, %	51 (39–57)	52 (42–59)	0.18
**Muscular function**			
ΔVO_2_/ΔWork-rate slope	9.89 (8.57–11.34)	9.24 (8.22–10.61)	0.12
**Others**			
VE/VO_2_ peak	35 (30–40)	36 (30–40)	0.10
RER peak	1.12 (0.10)	1.23 (0.12)	<0.01
VO_2_ AT, ml/kg/min	12.36 (3.86)	13.82 (4.86)	0.01

*Data are presented as the means (SD), medians (IQR), or n (%).*

*CPET, cardiopulmonary exercise testing; ALS, amyotrophic lateral sclerosis; VO_2_, oxygen consumption; HR, heart rate; SBP, systolic blood pressure; DBP, diastolic blood pressure; VE, minute ventilation; VCO_2_, carbon dioxide production; BR, breathing reserve; RER, respiratory exchange ratio; AT, anaerobic threshold; IQR, interquartile range.*

### Relationships of Cardiopulmonary Exercise Testing Variables With Disease Characteristics in Amyotrophic Lateral Sclerosis

**FIGURE 3:**
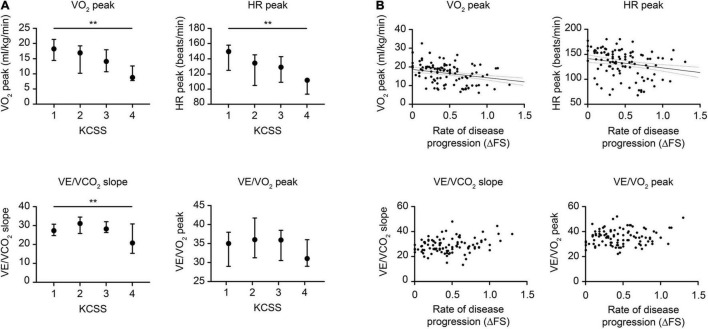
Changes in typical CPET variables relative to disease severity and disease progression in ALS. **(A)** Changes in the CPET relative to disease severity as graded by the KCSS. **(B)** Changes in the CPET relative to disease progression scored by the ΔFS. Typical CPET changes included (a) overall exercise capacity, represented by the VO_2_ peak, (b) cardiovascular function, represented by HR peak, (c) pulmonary function, represented by VE/VCO_2_ slope, and (d) breathing economy, represented by the VE/VO_2_ peak. The *p*-value is based on the Kruskal-Wallis test and Spearman’s correlation. Vertical lines indicate medians (IQR). ALS, amyotrophic lateral sclerosis; CPET, cardiopulmonary exercise testing; ΔFS, (48-[ALSFRS-R])/disease duration from symptom onset to the CPET assessment; ALSFRS-R, ALS Functional Rating Scale-Revised; KCSS, King’s College Staging System; VO_2_, oxygen consumption; HR, heart rate; VE, minute ventilation; VCO_2_, carbon dioxide production; IQR, interquartile range. **p < 0.01.

The changes in CPET variables in patients with different disease severities and rates of disease progression are shown in Figure 3. Intergroup comparisons were conducted when patients were categorized by KCSS into different levels of disease severity at assessment (Figure 3A). The VO_2_ peak and HR peak were significantly decreased in patients with more severe impairment (*p* < 0.001 and *p* = 0.007, respectively). With increasing disease severity, VE/VCO_2_ slope significantly increased first but later decreased (*p* = 0.015). In addition, no significant changes in the VE/VO_2_ peak were observed. Regarding the relationships between CPET variables and the rate of disease progression (ΔFS), correlation analyses were conducted (Figure 3B). The VO_2_ peak and HR peak were significantly decreased as the rate of progression increased (both *p* < 0.001). However, no significant relationships with the VE/VCO_2_ slope or VE/VO_2_ peak were observed. We also compared the CPET parameters in patients with and without daily use of riluzole and found no significant differences (Supplementary Table 2).

Correlation analyses between typical CPET variables and other disease characteristics of ALS are shown in Table 3. For the overall exercise capacity, the VO_2_ peak showed significantly positive correlations with FVC and the ALSFRS-R score (both *p* < 0.001) while significantly negative correlations with the LMN score (*p* = 0.016). For cardiovascular function, HR peak also showed significant relationships with BMI, FVC, and the ALSFRS-R score (*p* = 0.039, *p* = 0.043, *p* < 0.001, respectively). For pulmonary function, increased VE/VCO_2_ slope was significantly correlated with the increased LMN score and the decreased ALSFRS-R score (*p* = 0.001 and *p* = 0.017, respectively). Besides, an increased VE/VO_2_ peak was significantly correlated with the increased LMN score and the decreased BMI (*p* = 0.039 and *p* = 0.002, respectively). No relationships between any CPET variable and the UMN score or the sNfL were observed. We further conducted the multiple linear regression analysis to assess the adjusted association of disease-related variables with the VO_2_ peak: VO_2_ peak ml/kg/min = −3.394 + 2.916 (sex; 0 = female, 1 = male) −0.081 age + 0.522ALSFRSR-R (*F* = 15.076, *p* < 0.001). Based on the *R*^2^, the model explained 30% of the variance in the VO_2_ peak, showing that the VO_2_ peak decreased with decreasing ALSFRS-R.

**TABLE 3:** Correlations of CPET variables with disease characteristics of ALS.

	Overall exercise capacity VO_2_ peak		Cardiovascular function HR peak		Pulmonary function VE/VCO_2_ slope		Breathing economy VE/VO_2_ peak	
	R	*p*	R	*p*	R	*p*	R	*p*
BMI	−0.042	0.498	−0.198	**0.039**	−0.046	0.638	−0.301	**0.002**
FVC	0.383	**<0.001**	0.212	**0.043**	0.204	0.056	0.165	0.118
ALSFRS-R	0.475	**<0.001**	0.405	**<0.001**	−0.233	**0.017**	−0.063	0.517
UMN	−0.122	0.207	−0.062	0.520	0.148	0.131	0.031	0.751
LMN	−0.229	**0.016**	−0.087	0.368	0.325	**0.001**	0.199	**0.039**
KCSS	−0.390	**<0.001**	−0.325	**0.001**	0.077	0.434	0.028	0.770
NfL	−0.344	0.079	−0.256	0.198	0.158	0.442	−0.045	0.825
ΔFS	−0.433	**<0.001**	−0.430	**<0.001**	0.138	0.162	−0.031	0.751

*The p-value is based on Spearman’s correlation analysis. p < 0.05 is indicated in bold.*

*CPET, cardiopulmonary exercise testing; ALS, amyotrophic lateral sclerosis; VO_2_, oxygen consumption; HR, heart rate; VE, minute ventilation; VCO_2_, carbon dioxide production; BMI, body mass index; FVC, forced vital capacity; ALSFRS-R, ALS Functional Rating Scale-Revised; UMN, upper motor neuron; LMN, lower motor neuron; KCSS, King’s College Staging System; NfL, neurofilament light chain; ΔFS, (48-[ALSFRS-R])/disease duration from symptom onset to the CPET assessment.*

### Prognostic Values of Cardiopulmonary Exercise Testing Variables in Amyotrophic Lateral Sclerosis

**FIGURE 4:**
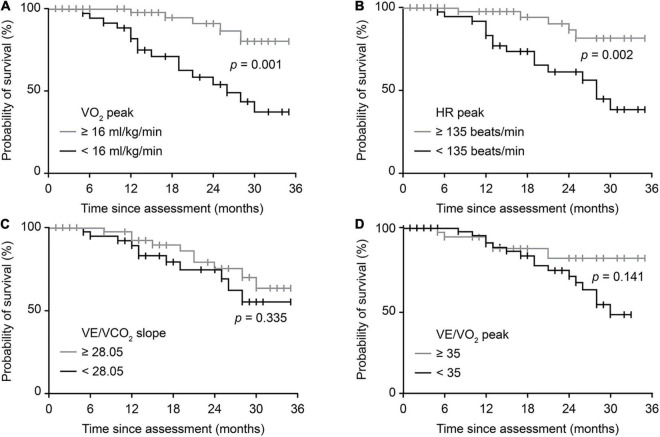
Survival probability predicted by typical CPET variables. Crude Kaplan-Meier curves during follow-up for **(A)** overall exercise capacity, represented by the VO_2_ peak, **(B)** cardiovascular function, represented by HR peak, **(C)** pulmonary function, represented by VE/VCO_2_ slope, and **(D)** breathing economy, represented by the VE/VO_2_ peak. The cutoff value for the VO_2_ peak was defined by previous studies. The cutoff values for the remaining three variables were based on the medians of patients in this study. |, censored patients. ALS, amyotrophic lateral sclerosis; CPET, cardiopulmonary exercise testing; VO_2_, oxygen consumption; HR, heart rate; VE, minute ventilation; VCO_2_, carbon dioxide production.

There were 21 endpoint events for patients with ALS throughout the study. Figure 4 shows the survival probability of patients with typically impaired CPET variables reflecting overall or relative function through univariable analysis (Kaplan-Meier survival curves with log-rank test). Patients with a VO_2_ peak < 16 ml/kg/min and an HR peak < 135 beats/min had significantly worse survival (*p* = 0.001, *p* = 0.002, respectively). However, no significant differences were identified using the VE/VCO_2_ slope or VE/VO_2_ peak. Univariate analyses for the Cox regression model are presented in Supplementary Table 3. Further multivariable Cox regression analysis (Table 4) identified that a spinal-onset phenotype (*p* = 0.014), an increased VO_2_ peak (*p* = 0.001), and an increased HR peak (*p* < 0.001) were independently associated with a better prognosis of ALS during follow-up (HR = 0.263, *p* = 0.014; HR = 0.839, *p* = 0.001; HR = 0.967, *p* < 0.001, respectively). In contrast, an increased VE/VO_2_ peak was a detrimental factor independently, indicating a worse survival outcome (HR = 1.137, *p* = 0.028).

**TABLE 4:** Prognostic factors associated with survival in multivariable Cox analysis.

	HR	95% CI	*p*-value
Age of onset, years	1.022	0.978–1.068	0.341
**Sex**			
Female	1.000		
Male	0.864	0.343–2.180	0.757
BMI, kg/m^2^	0.917	0.794–1.059	0.239
**Site of onset**			
Bulbar	1.000		
Spinal	0.263	0.091–0.763	0.014
VO_2_ peak, ml/kg/min^a^	0.839	0.757–0.930	0.001
HR peak, beats/min^b^	0.967	0.950–0.985	<0.001
VE/VCO_2_ slope^b^	0.962	0.905–1.024	0.225
VE/VO_2_ peak^b^	1.137	1.014–1.274	0.028

*The multivariable models included previously established prognostic indicators for ALS and CPET variables (a included the overall CPET parameter reflecting whole-body exercise capacity; b included the three system-specific CPET parameters reflecting cardiovascular, pulmonary, and breathing economy separately).*

*VO_2_, oxygen consumption; HR, heart rate; VE, minute ventilation; VCO_2_, carbon dioxide production; BMI, body mass index.*

## Discussion

This study performed comprehensive CPET measurements to investigate exercise physiology impairments in patients with ALS compared with age- and sex-matched healthy controls. This is a pioneering study to reveal the predictive values of CPET parameters for disease severity and prognosis of ALS in a prospective cohort. We provided comprehensive evidence for the application of CPET in ALS: our results quantify the significant decrease in exercise capacity in patients with ALS compared with strictly matched controls, including the overall VO_2_ peak and the system-targeted HR peak and VE/VCO_2_ slope. Furthermore, CPET parameters were significantly associated with disease severity scores, which showed the potential use of CPET as an objective marker for non-invasively quantifying disease impairment. Finally, typical CPET variables could distinguish patients with different prognoses well since the VO_2_ peak, HR peak, and VE/VO_2_ peak emerged as independent predictors of survival in ALS. Thus, CPET could provide novel evaluations from the perspective of exercise physiology in ALS and benefit not only for intervention timing in the clinic but also for stratification of patients in scientific research.

Among the CPET parameters, the VO_2_ peak was identified as the most significant parameter of ALS in our study. As a well-recognized variable to represent the exercise capability, the VO_2_ peak quantitatively reflects the exercise capacity of the whole body (Guazzi et al., 2012, 2016). We thoroughly identified the prevalence and range of VO_2_ peak alterations in ALS compared with strictly matched controls (Figure 2). The significantly higher frequency of impaired exercise capacity in patients with ALS (44.95%) addressed the extensive functional decline. Furthermore, in our supplementary analysis, the VO_2_ peak showed a significant decline in patients with a disease duration less than 12 months compared with healthy controls [16.23 (5.62) vs. 22.26 (7.09); *p* < 0.01; Supplementary Table 4]. Thus, the remarkable impairment in early phase patients emphasizes the sensitivity of exercise capacity for early disease impairment. Additionally, the positive correlation and survival analyses revealed the meaningful value of the VO_2_ peak in the clinical practice of ALS. The significant associations between the VO_2_ peak and most established clinical parameters of ALS (Table 2) suggest that it can be used as a comprehensive, objective, and quantitative measurement for monitoring disease severity and progression in ALS. As an indicator reflecting the whole-body physiological response, the VO_2_ peak comprehensively summarizes the overall capacity for exercise affected by multiple systems (Guazzi et al., 2017). In addition, it is more objective as a quantitative index than traditional parameters based on self-reported symptoms (Fournier et al., 2020). Finally, our consistently positive results in univariable and multivariable survival analyses also highlight the prognostic role of the VO_2_ peak for better outcomes in ALS. Given its repeatability and modifiability, the VO_2_ peak has been used as a primary endpoint for non-invasively assessing the response to the treatment of physical exercise in several randomized clinical trials (Shulman et al., 2013; Wallace et al., 2019). Since improvements in the VO_2_ peak after intervention have been observed in patients with inclusion body myositis and Charcot-Marie-Tooth disease, longitudinal studies on CPET in ALS are needed in the future to explore potential treatments from the perspective of exercise capacity to improve survival (Wallace et al., 2019).

In our study, CPET also identified the exercise physiology impairments in different systems to support the multisystem impairments in ALS from a functional perspective. We established direct relationships between the system-specific CPET parameters and the pathophysiological mechanism of the different systems affected in ALS (Guazzi et al., 2012, 2016). Additionally, we revealed that the decline in overall exercise capacity had multifaceted origins involving cardiovascular, pulmonary, and muscular impairment in ALS.

Conflicting data have been reported regarding cardiovascular disorders in ALS (Pereira et al., 2021). Our study identified the cardiac involvement of ALS with abnormal autonomic nervous function. We observed a significantly higher HR at rest in patients, which supports the continuous impairment of the sympathetic nervous system in ALS. Tanaka et al. (2013) recognized similar sympathetic hyperactivity with cardiac [^123^I] MIBG scintigraphy and reasoned that they were related to sudden cardiac arrest in ALS. However, HR at peak exercise was significantly limited among our patients, which implies a dysregulation of the sympathetic nervous system in accommodating higher exercise levels. Since no significant difference was shown in HR recovery after exercise, we supposed that the parasympathetic nervous system was relatively reserved in ALS. Thus, cardiovascular impairment in ALS does not resemble the CPET pattern typical of infiltrative cardiomyopathy with decreased HR recovery (Patel et al., 2014). Furthermore, our positive correlation and survival analyses recognized that the HR peak was similar to the VO_2_ peak for disease evaluation. This emphasizes the significant influence of the blunted HR response on overall exercise capacity in ALS.

For the pulmonary system, the significantly increased VE/VCO_2_ slope suggests a ventilatory limitation in patients with ALS healthy controls in our study. Recognized as a measure of ventilatory efficiency, the VE/VCO_2_ slope reflects the capacity of CO_2_ elimination during the whole exercise process (Neder et al., 2017). The weakened respiratory muscles in ALS are known to cause decreased breathing activity, leading to ventilatory insufficiency (van Es et al., 2017). An increased VE/VCO_2_ slope is typical in chronic obstructive pulmonary disease, another restrictive ventilatory disorder with a similar pathophysiological mechanism of pulmonary dysfunction to that in ALS (Neder et al., 2017). However, no significant difference in the survival of patients with different VE/VCO_2_ slopes was observed in our study. We attributed this to the relatively mild respiratory dysfunction of patients in our cohort as the median value of the VE/VCO_2_ slope in our patients did not reach the recommended cutoff value for ventilatory limitation (>30) (Guazzi et al., 2012). Further studies including patients with more severe respiratory insufficiency are required.

Regarding the muscular system, our results revealed no significant dysfunction of muscular oxygen utilization in patients with ALS compared with healthy controls. Considering that muscle dysfunction is indirect and secondary to neuronal impairment in ALS, it is reasonable that our CPET pattern is different from that seen in mitochondrial myopathies (Jeppesen et al., 2003). The VE/VO_2_ peak was a comprehensive indicator of breathing economy. In the muscular disease with scarce impairment in the cardiopulmonary system, the higher VE/VO_2_ peak has been reported to directly reflect the lower muscle oxidative capacity in the patients with mitochondrial myopathy compared with controls (Taivassalo et al., 2003). Besides, increased VE/VO_2_ peak has also been identified in cardiopulmonary diseases for ventilatory inefficiency (i.e., heart failure) (Mejhert et al., 2002). Since ALS is a complex disease with the involvement of multiple systems, the interpretation of the VE/VO_2_ peak could be a comprehensive indicator for impairments in both cardiopulmonary and muscular systems. Moreover, the significant correlation of clinical survival supported its potential use as a prognostic indicator in ALS (Table 4). Further studies including the detection of plasma lactate and biopsies of muscle tissues are needed to provide more direct evidence of muscular mitochondrial abnormalities in exercise intolerance of ALS.

Neurofilament light chain (NfL) was not significantly associated with typical CPET parameters in this study. This could be due to different pathogenic mechanisms represented by the two variables in ALS. NfL, the main by-product of neuroaxonal breakdown, mainly represents the degree of axonal damage and reflects the neurodegeneration process of ALS (Lu et al., 2015). In contrast, abnormal CPET parameters, the direct representative of exercise physiology impairments, mainly reflect the dysregulation of multiple systems under the exercise stressor (Guazzi et al., 2017). The negative correlations could be reasonable since the parameters stand for the pathophysiology of different dimensions in ALS. Further studies with a larger sample size and more elaborate study design are needed to get a better understanding of the issue.

This study has several limitations. First, since the standard CPET requires the adaptability to pedal the apparatus for the cycling test, we enrolled patients at the time of diagnosis to ensure functional availability. This might limit the application in late-stage patients with severe impairments. Furthermore, given the characteristics of CPET, we suggest conducting this evaluation in the early stage of patients with ALS. Second, this study was conducted in patients with sporadic ALS, independent replication of this study in those with familial ALS with a specific genetic background is essential to support a broader application of CPET in ALS. In addition, although this preliminary study first explored the clinical value of CPET in ALS, the present investigation was conducted in a single center with potential referral bias. Thus, multicenter studies involving larger numbers of participants are needed to confirm our findings in the future.

In summary, through the comprehensive assessment of CPET, we quantified the common and significant exercise physiology impairments in patients with ALS compared with matched controls. CPET variables were significantly associated with functional scores reflecting disease severity and progression. We demonstrated that patients with a higher VO_2_ peak, HR peak, and a lower VE/VO_2_ peak had better survival in ALS. Our findings of exercise physiology impairments also highlight the potential use of CPET in ALS for the non-invasive, quantitative, and objective evaluation of disease severity and clinical prognosis.

## Data Availability Statement

The raw data supporting the conclusions of this article will be made available by the authors, without undue reservation.

## Ethics Statement

The studies involving human participants were reviewed and approved by the institutional Ethics Committee of the Peking University Third Hospital. The patients/participants provided their written informed consent to participate in this study.

## Author Contributions

JH, DF, JF, WZ, and CR: concept and design. JH, JF, and WZ: drafting of the manuscript. DF, WZ, and CR: critical revision of the manuscript for important intellectual content. NL, LC, JH, and JF: statistical analysis. DF and JH: obtain funding. CR, PL, DL, LZho, and LC: administrative, technical, and material support. JH, DF, WZ, JF, CR, and PL: supervision. All authors: acquisition, analysis, or interpretation of data.

## Conflict of Interest

The authors declare that the research was conducted in the absence of any commercial or financial relationships that could be construed as a potential conflict of interest.

## Publisher’s Note

All claims expressed in this article are solely those of the authors and do not necessarily represent those of their affiliated organizations, or those of the publisher, the editors and the reviewers. Any product that may be evaluated in this article, or claim that may be made by its manufacturer, is not guaranteed or endorsed by the publisher.
