# Supplementary material for: Exercise Physiology Impairments of Patients With Amyotrophic Lateral Sclerosis: Cardiopulmonary Exercise Testing Findings
Source: Front Physiol. 2022 Mar 14;13:792660. doi: 10.3389/fphys.2022.792660 (PMC8967153; doi:10.3389/fphys.2022.792660)
Supplement: Supplementary file 1 [file Table_1.DOCX]

**Supplementary Table 1.** Standardized routine records of detailed demographic and clinical information for ALS in Peking University Third Hospital

| **Measurements** | **Definitions** |
| --- | --- |
| \| **Assessments of the first visit** \| \| --- \| | |
| Demographic information | • Sex, age, date of birth, ethnicity  • Dominant hand  • Education, marriage, medical insurance, occupation |
| Lifestyle information | • Food diary, mental status, physical activity, quality of life, environmental exposure (toxicant) |
| Medical history | • Past medical history, family history, trauma history, reproductive history |
| Diagnosis information | • Date of diagnosis (fist and confirmed), delay of diagnosis, age of diagnosis, level of diagnosis  • Hospital of diagnosis, type of clinic (outpatient or inpatient)  • Diagnosis of genetic test  • Diagnosis of clinical phenotype |
| Variables at clinical onset | • Date of onset, age of onset, site of onset  • Symptoms of onset (motor symptoms/cognitive symptoms/nonmotor symptoms)  • Physical examinations at onset (upper motor impairment/lower motor impairment/cognitive impairment)  • Electromyography test at onset  • ALS-FRS-R  • Height, weight, BMI  • Lung function (FVC, FVC%) |
| **Assessments of follow-up visits** | |
| Variables during clinical progression | • Date of progression, progression of affected sites  • Progression of symptoms (motor symptoms/cognitive symptoms/nonmotor symptoms)  • Progression of physical examination results  • Progression of electromyography test results  • ALS-FRS-R and ΔALS-FRS-R  • Height, weight, BMI and Δheight, weight, BMI  • Lung function (FVC, FVC%) and Δlung function (FVC, FVC%) |
| Treatment information | • Use of medications (riluzole/traditional Chinese medicine/other medications)  • Respiration assistance (use of noninvasive ventilation)  • Diet assistance (use of intubation/gastrostomy)  • Other (e.g., rehabilitation, stem cell treatment, gene therapy, etc.)  • Date of start and end of use  • Details of use |
| Endpoint events | • Death due to any cause  • Tracheotomy  • Date of the event  • Reasons for the event |

ALS-FRS-R, amyotrophic lateral sclerosis functional rating scale-revised; BMI, body mass index; FVC, forced vital capacity; Δ=rate of decline from onset to the time of assessment

**Supplementary Table 2. CPET assessments of ALS patients** **with and without daily use of riluzole**

|  | With riluzole, n=71 | Without riluzole, n=38 | *P* Value |
| --- | --- | --- | --- |
| Overall exercise capacity | | | |
| VO_2_ peak, ml/kg/min | 16.27 (5.27) | 15.94 (5.80) | 0.763 |
| VO_2_ peak ≥16 ml/kg/min, no. (%) | 37 (52.1%) | 23 (60.5%) | 0.400 |
| VO_2_ peak <16 ml/kg/min, no. (%) | 34 (47.9%) | 15 (39.5%) |  |
| Cardiovascular function | | | |
| HR rest, beats/min | 88 (78-98) | 84 (75-94) | 0.227 |
| SBP rest, mm Hg | 129 (119-138) | 125 (112-134) | 0.191 |
| DBP rest, mm Hg | 80 (74-88) | 78 (74-83) | 0.316 |
| HR peak, beats/min | 137 (117-155) | 132 (106-152) | 0.244 |
| SBP peak, mm Hg | 164 (141-194) | 166 (142-184) | 0.499 |
| DBP peak, mm Hg | 86 (80-96) | 86 (77-95) | 0.810 |
| HR recovery, beats/min | 23 (19-31) | 24 (16-32) | 0.698 |
| Pulmonary function | | | |
| VE/VCO_2_ slope | 28.39 (25.40-32.16) | 27.63 (24.00-32.21) | 0.622 |
| BR, % | 49 (40-57) | 56(38-58) | 0.265 |
| Muscular fuction |  |  |  |
| ∆VO_2_/∆Work-rate slope | 9.91 (8.58-11.36) | 9.13 (8.17-10.60) | 0.478 |
| Others |  |  |  |
| VE/VO_2_ peak | 36 (30-40) | 35 (30-39) | 0.726 |
| RER peak | 1.13 (0.10) | 1.11 (0.10) | 0.547 |
| VO_2_ AT, ml/kg/min | 12.35 (3.49) | 12.39 (4.52) | 0.952 |

Data are presented as the means (SD), medians (IQR) or n (%).

Abbreviations: CPET, cardiopulmonary exercise testing; ALS, amyotrophic lateral sclerosis; BMI, body mass index; VO_2_, oxygen consumption; HR, heart rate; SBP, systolic blood pressure; DBP, diastolic blood pressure; VE, minute ventilation; VCO_2_, carbon dioxide production; BR, Breathing Reserve; RER: respiratory exchange ratio; AT: anaerobic threshold; IQR, interquartile range.

**Supplementary Table 3. Prognostic Factors Associated with Survival in Univariable Cox Analysis**

|  | HR | 95% CI | *P* Value |
| --- | --- | --- | --- |
| Age of onset, y | 1.037 | 0.995-1.081 | 0.086 |
| Sex |  |  |  |
| Female | 1.000 |  |  |
| Male | 0.526 | 0.223-1.244 | 0.143 |
| BMI, kg/m^2^ | 0.957 | 0.831-1.102 | 0.543 |
| Site of onset |  |  |  |
| Bulbar | 1.000 |  |  |
| Spinal | 0.357 | 0.143-0.889 | 0.027 |
| VO_2_ peak, ml/kg/min | 0.876 | 0.808-0.950 | 0.001 |
| HR peak, beats/min | 0.976 | 0.962-0.990 | 0.001 |
| VE/VCO_2_ slope | 0.955 | 0.895-1.019 | 0.161 |
| VE/VO_2_ peak | 1.023 | 0.952-1.099 | 0.542 |

The univariable models included previously established prognostic indicators for ALS and CPET variables.

Abbreviations: VO_2_, oxygen consumption; HR, heart rate; VE, minute ventilation; VCO_2_, carbon dioxide production; BMI, body mass index.

**Supplementary Table 4.** **CPET assessments of controls and ALS patients with disease duration less than 12 months**

|  | ALS, n=55 | Control, n=150 | *P* Value |
| --- | --- | --- | --- |
| Overall exercise capacity | | | |
| VO_2_ peak, ml/kg/min | 16.23 (5.62) | 22.26 (7.09) | <0.01 |
| VO_2_ peak ≥16 ml/kg/min, no. (%) | 32 (58.20%) | 136 (90.67%) | <0.01 |
| VO_2_ peak <16 ml/kg/min, no. (%) | 23 (41.80%) | 14 (9.33%) |  |
| Cardiovascular function | | | |
| HR rest, beats/min | 87 (78-97) | 83 (73-90) | 0.02 |
| SBP rest, mm Hg | 129 (121-139) | 122 (113-138) | 0.02 |
| DBP rest, mm Hg | 80 (76-86) | 78 (70-86) | 0.09 |
| HR peak, beats/min | 135 (111-153) | 148 (135-164) | <0.01 |
| SBP peak, mm Hg | 168 (143-190) | 166 (145-191) | 0.72 |
| DBP peak, mm Hg | 86 (79-95) | 83 (77-90) | 0.12 |
| HR recovery, beats/min | 22 (17-30) | 25 (19-31) | 0.11 |
| Pulmonary function | | | |
| VE/VCO_2_ slope | 27.38 (24.28-32.45) | 26.72 (24.37-29.58) | 0.22 |
| BR, % | 49 (37-57) | 52 (42-59) | 0.20 |
| Muscular fuction |  |  |  |
| ∆VO_2_/∆Work-rate slope | 9.70 (8.46-11.20) | 9.24 (8.22-10.61) | 0.14 |
| Others |  |  |  |
| VE/VO_2_ peak | 34 (30-39) | 36 (30-40) | 0.02 |
| RER peak | 1.13 (0.10) | 1.23 (0.12) | <0.01 |
| VO_2_ AT, ml/kg/min | 12.53 (3.66) | 13.82 (4.86) | 0.07 |

Data are presented as the means (SD), medians (IQR) or n (%).

Abbreviations: CPET, cardiopulmonary exercise testing; ALS, amyotrophic lateral sclerosis; BMI, body mass index; VO_2_, oxygen consumption; HR, heart rate; SBP, systolic blood pressure; DBP, diastolic blood pressure; VE, minute ventilation; VCO_2_, carbon dioxide production; BR, Breathing Reserve; RER: respiratory exchange ratio; AT: anaerobic threshold; IQR, interquartile range.
